# Mapping of the Underlying Neural Mechanisms of Maintenance and Manipulation in Visuo-Spatial Working Memory Using An n-back Mental Rotation Task: A Functional Magnetic Resonance Imaging Study

**DOI:** 10.3389/fnbeh.2016.00087

**Published:** 2016-05-06

**Authors:** Gemma Lamp, Bonnie Alexander, Robin Laycock, David P. Crewther, Sheila G. Crewther

**Affiliations:** ^1^Department of Psychology and Counselling, School of Psychology and Public Health, LaTrobe UniversityMelbourne, VIC, Australia; ^2^Centre for Human Psychopharmacology, Swinburne UniversityMelbourne, VIC, Australia

**Keywords:** working memory, maintenance, manipulation, fMRI, n-back, mental rotation

## Abstract

Mapping of the underlying neural mechanisms of visuo-spatial working memory (WM) has been shown to consistently elicit activity in right hemisphere dominant fronto-parietal networks. However to date, the bulk of neuroimaging literature has focused largely on the maintenance aspect of visuo-spatial WM, with a scarcity of research into the aspects of WM involving manipulation of information. Thus, this study aimed to compare maintenance-only with maintenance and manipulation of visuo-spatial stimuli (3D cube shapes) utilizing a *1*-back task while functional magnetic resonance imaging (fMRI) scans were acquired. Sixteen healthy participants (9 women, *M* = 23.94 years, *SD* = 2.49) were required to perform the *1*-back task with or without mentally rotating the shapes 90° on a vertical axis. When no rotation was required (maintenance-only condition), a right hemispheric lateralization was revealed across fronto-parietal areas. However, when the task involved maintaining and manipulating the same stimuli through 90° rotation, activation was primarily seen in the bilateral parietal lobe and left fusiform gyrus. The findings confirm that the well-established right lateralized fronto-parietal networks are likely to underlie simple maintenance of visuo-spatial stimuli. The results also suggest that the added demand of manipulation of information maintained online appears to require further neural recruitment of functionally related areas. In particular mental rotation of visuospatial stimuli required bilateral parietal areas, and the left fusiform gyrus potentially to maintain a categorical or object representation. It can be concluded that WM is a complex neural process involving the interaction of an increasingly large network.

## Introduction

Working memory (WM) was originally defined as short-term retention of information (Miller et al., 1960). Historically WM has generally been considered as a static state of information, with an external attentional mechanism that allocates the storage of stimulus representations (Baddeley and Hitch, 1974). Over the years however, the construct has evolved to a much more sophisticated concept involving constant attention and incorporation of aspects of data manipulation with respect to behavioral goals (Postle, 2006). One-to-one mapping of cognitive models of WM to neural mechanisms has been problematic (D'Esposito, 2007) and often results in compensatory models which have not been able to be systematically verified (Baddeley, 2003).

A general consensus has been reached that there is involvement of fronto-parietal networks in WM tasks (Wager and Smith, 2003; Owen et al., 2005; Rottschy et al., 2012). However, while the importance of this fronto-parietal network in WM is demonstrated throughout the literature, the fractionation of functional roles across these regions has yet to be understood and agreed upon. Many authors accept that fronto-parietal activation may represent attentional directives during WM maintenance (Gazzaley and Nobre, 2012), and that these areas activated during WM maintenance also show activity considered to be related particularly to spatial attention (Ikkai and Curtis, 2011). However, WM may not be restricted only to fronto-parietal sites.

Stimulus-specific hemispheric lateralization has been suggested, with a left hemisphere (LH) dominance in frontal areas thought to represent “verbal” WM (Wager and Smith, 2003) or “categorical” representations of stimuli (van der Ham et al., 2009) and right hemisphere (RH) dominance for “visual” tasks (Owen et al., 2005), specifically “visuo-spatial” (Vogel et al., 2003; van der Ham et al., 2009). Different stimulus categories also appear to result in a dorsal/ventral delineation in areas activated. Object identity based WM has been localized to ventral areas such as the inferior temporal lobe (Wager and Smith, 2003) and the inferior frontal gyrus (Jackson et al., 2011). Conversely WM involving visuo-spatial stimuli has been localized to more dorsal areas of the brain, such as the parietal lobe (Wager and Smith, 2003) and superior frontal lobe (Jackson et al., 2011).

WM research has also revealed strong activation in the superior parietal lobe (SPL), regardless of attentional demand (Culham and Kanwisher, 2001), with the degree of inferior parietal lobe (IPL) activation dependent on increasing load (Harrison et al., 2010). Examining attentional processes however requires acknowledgment of the division into two main processes: top down which refers to cognitively controlled, goal-directed attention, and bottom up which is captured exogenously by sensory stimuli. While bottom up attention has been suggested to be right lateralized and localized in the temporo-parietal and ventral frontal cortices, top down attention has been suggested to require both hemispheres and is commonly attributed to the dorsal parietal and frontal cortices (Corbetta and Shulman, 2002), with a major emphasis placed on the SPL and precuneus (Behrmann et al., 2004).

Studies of cognitive tasks have led toward a more network-based approach to investigating the neural basis of cognition, with areas shown to activate and deactivate together suggested to underlie cognitive processes. A recent study by Menon and Uddin (2010) examined the involvement of three important networks in WM: the default mode network (DMN), including the posterior cingulate cortex (PCC), and ventro-medial PFC; the central executive network, including the dorso-lateral prefrontal cortex (DLPFC) and posterior parietal cortex (PPC); and the salience network, including the anterior cingulate cortex (ACC) and anterior insula. The concurrent activation of the central executive network and salience network, together with the deactivation of the DMN, was suggested to underlie cognitively demanding tasks such as WM. Hence, with previous WM research finding similar arrays of sites activated (Wager and Smith, 2003; Owen et al., 2005; Rottschy et al., 2012), it becomes clear there is a need to view cognitive processes as having multiple underlying networks of both activation and relative deactivation.

A large portion of the research into visuo-spatial WM utilizes maintenance-centric WM tasks with little to no manipulation involved. For example, the most common paradigm used to examine visuo-spatial WM involves maintaining a location in temporary storage then recalling that location upon demand (Awh et al., 1999, 2000; Kubler et al., 2003; Levin et al., 2005; Postle et al., 2005; Berryhill and Olson, 2008; McNab et al., 2008; Hartley and Sikora, 2010). This task is occasionally expanded to include maintenance and manipulation of locations (Glahn et al., 2002), maintenance of a color word followed by identification of the location of a circle the same color as the word (Munneke et al., 2010), or targeting of shapes with a pre-specified orientation (Mayer et al., 2007). Other studies have examined visuo-spatial processing outside of WM, using, for example, a line bisection task (De Schotten et al., 2011) or rotation of blocks (Schöning et al., 2007), and implicate the involvement of similar areas located in simple visuo-spatial WM tasks. However, research into location monitoring n-back tasks is sparse and other styles of “visuo-spatial” n-back tasks are lacking, suggesting further research in visuo-spatial WM is required.

In particular, WM processes required for tasks utilizing visuo-spatial stimuli have historically been associated with mental rotation tasks based on the design of Shepard and Metzler (1971). Mental rotation refers to the cognitive manipulation of objects rather than physical rotation. In literature exploring the neuroanatomical basis of mental rotation, there has been continuing debate regarding the hemispheric lateralization of the task, with most studies acknowledging a RH advantage (Corballis, 1997). Although RH specificity was originally thought to be primarily found in men, more recent studies have shown both women and men show activation of the right SPL and bilateral occipital cortices (Halari et al., 2006), with substantial parietal involvement (Jordan et al., 2001; Olson and Berryhill, 2009) that becomes more dominant during more complicated rotations (Just et al., 2001).

A psychophysical study of mental rotation and WM by Hyun and Luck (2007) suggested that identification and mental rotation of letters appeared to use object but not spatial WM. When asked to hold an object in memory while simultaneously rotating a letter, performance on the task declined. However, minimal interference was apparent when participants held a location in memory while simultaneously rotating letters (Hyun and Luck, 2007). This observation can partially be explained by an fMRI study that compared simultaneous mental rotation of 2-D and 3-D blocks in a WM task. The 3-D cube rotation was associated with DLPFC and medial frontal activation, while 2-D spatial matrix processing (not involving mental rotation) activated the right inferior prefrontal cortex (PFC), bilateral IPL, and left SPL (Suchan et al., 2006). However, no neuroimaging studies to date have examined mental rotation and WM simultaneously to dissociate maintenance from manipulation.

Maintenance-only compared to maintenance plus manipulation has been examined in a verbal WM task (Champod and Petrides, 2010). Participants were required to either simply remember a list of words (maintenance) or to simultaneously re-order the list (maintenance plus manipulation). In so doing the different tasks dissociated activation during maintenance from that for manipulation, finding maintenance to be subserved by the mid-DLPFC, and maintenance plus manipulation associated with activation in the inferior parietal sulcus (IPS). It remains unknown whether such a dissociation of frontal areas subserving maintenance and parietal areas underlying manipulation would be replicated in a visuospatial WM task.

The current study also aimed to contrast a maintenance-only WM task with a maintenance plus manipulation WM task, while utilizing spatial stimuli and mental rotation as the manipulation component. A simple *1*-back design WM task using classical visuo-spatial stimuli of 3-D block shapes constituted the maintenance-only task. The same design and stimuli was used for a second task, but required the participant to concurrently maintain the first image presented and then rotate the image to judge whether the second image presented was congruent and rotated or different to the original. It was hypothesized that the maintenance-only visuospatial *1*-back task would activate a RH dominant network of sites in the frontal, parietal, and insula areas. With the added demand of manipulation (mental rotation), it was hypothesized that activation would become more dorsal, with more parietal involvement.

## Methods

### Participants

Sixteen right-handed adults (9 women) aged 18–27 (*M* = 23.94 years, *SD* = 2.49) participated after being screened for normal color vision and having no history of neurological disorder or disease. Research was undertaken with the informed and written consent of each participant, with the approval of the Swinburne University and La Trobe University Human Ethics Committees, and in compliance with national legislation and the Code of Ethical Principles for Medical Research Involving Human Subjects of the World Medical Association.

### Working memory tasks

**Figure 1:**
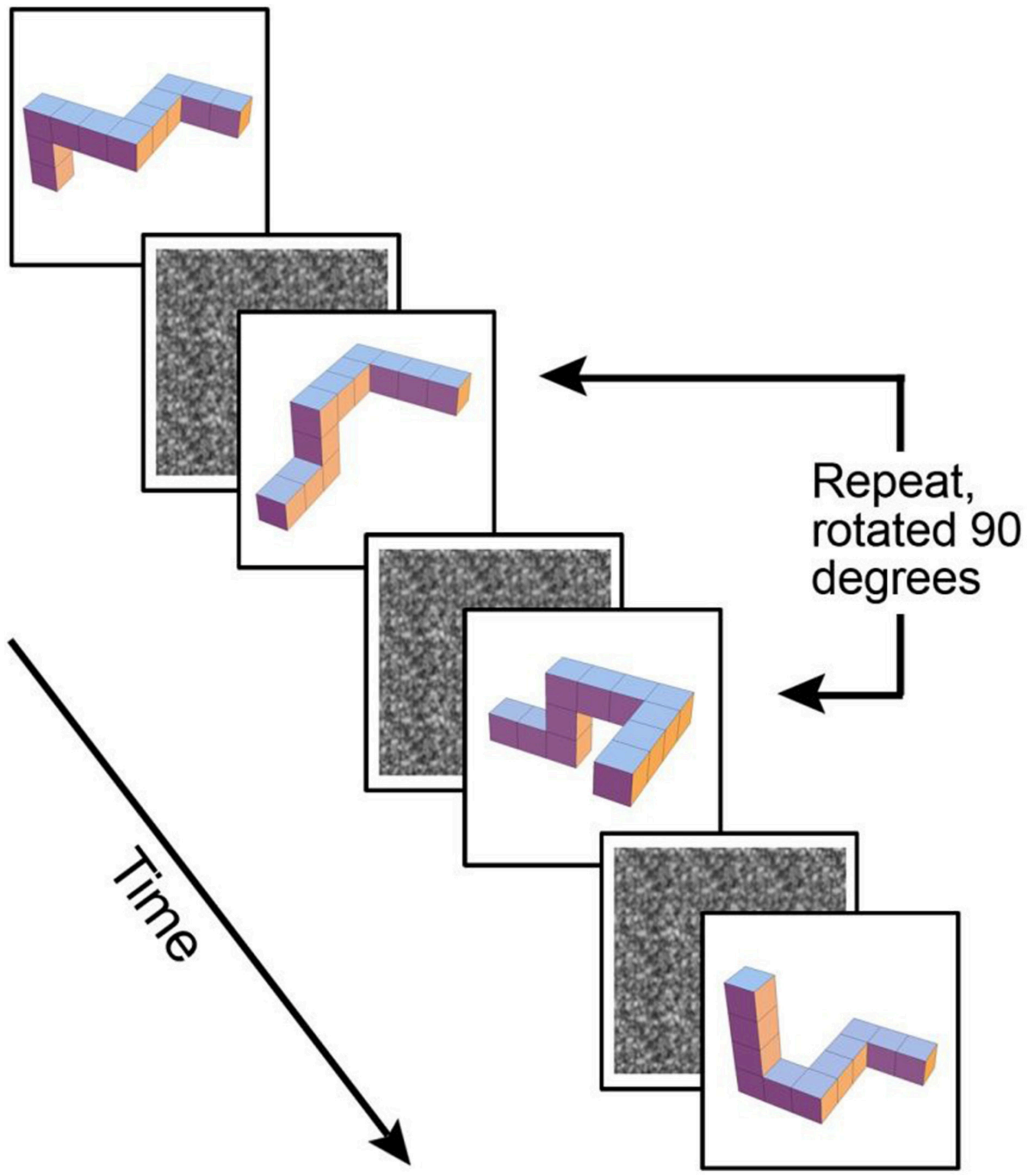
**Example of maintenance plus manipulation paradigm, 3-D block images rotated 90° on a vertical axis**.

The first maintenance only WM *1*-back task involved a simple identical repeat or non-repeat design of 3-D block shapes. The second maintenance plus manipulation WM *1*-back task involved the same 3-D cubes, with the additional requirement to mentally rotate the shapes 90° on a vertical axis in order to determine if they were a repeated shape (see Figure 1).

For both tasks, stimuli were presented for 0.5 s followed by a masking white noise stimulus (see Figure 1) for a randomized time interval, averaging 2.6 s and then followed by the next stimulus. Participants were asked to indicate whether each stimulus was a repeat of the previous shape. Two-alternative forced choice button responses were used to indicate either presence or absence of a repeat stimulus. Responses were made via a magnet-safe response box with two keys on it placed under participants' right hand. Each WM task used a blocked design, comprised of eight alternating blocks: four blocks of the WM task, and four blocks of a baseline. During baseline the participants remained active to control for motor responses. The same gray block shape was repeated five times and participants remained active, pressing the “repeat” button for each gray block shape. The task involved 132 trials in total, with 28 trials in an active block. In approximately 35% of the active trials a target match occurred, with a maximum of three targets in a row at one time. Each task was run as a separate scan during the same MRI session, with the maintenance-only task always presented before the maintenance plus manipulation task. Participants were familiarized with the tasks outside the scanner prior to scanning.

WM tasks were presented on a Macintosh iMac running Mac OS 9.1 (Apple Computer, Inc.), with a PowerPC G3 processor. Authorware Professional software for Macintosh version 2.2.0 US (Macromedia, Inc.) was used to run the tasks and created data text files recording reaction times and accuracy. Accuracy was recorded in terms of hit, miss, false alarm and correct rejection for each trial. Immediately following the scan participants were asked how difficult they found each task, on a Likert scale of 1–5, “1” representing easy and “5” representing difficult. Participants were also asked how they performed the mental rotation of the cubes, with responses coded as using a verbal strategy (subvocally describing the shapes and rotation) or a visual strategy (visualizing the blocks and rotating them visually in their mind).

### fMRI procedure

Image acquisition was performed using a General Electric Signa 3 Tesla MRI scanner at the Brain Research Institute, Melbourne. Both WM tasks were displayed via a video projector onto a screen inside the magnet room, and viewed through a mirror mounted above participants' eyes. The image subtended 8° vertically and 12° horizontally when viewed from an effective distance of 4 m. Participant responses and reaction time were recorded through a response box that fed back into the stimuli presentation computer running Authorware. Participants were asked to press the right button for “same” and the left button for “different.” T2^*^ weighted functional images were acquired during WM task performance (TR = 2500 ms, TE = 40 ms, flip angle = 60°, FOV = 240 mm, matrix = 96 × 96, 26 slices, 1 mm spacing, in plane resolution = 2.5 mm). High resolution 3-D T1 weighted anatomical images were also acquired (slices = 124, thickness = 1.4 mm, FOV = 240 mm, matrix = 512 × 256, flip angle = 20°).

### Data analysis

Psychophysical data was entered into SPSS [Computer Software] (SPSS Inc. Released 2009. PASW Statistics for Windows, Version 18.0. Chicago: SPSS Inc.) to determine subjective difficulty ratings, reaction time and accuracy on the task (in terms of correct identification of a repeat stimulus). Group means, standard deviations and range were calculated for each task separately. The relationship between these measures was then assessed using a Spearman's Rho, due to the mix of interval and ordinal data.

Preprocessing and analysis of fMRI data was performed using Statistical Parametric Mapping (SPM; Version 8) [Computer Software] (2014; retrieved from http://www.fil.ion.ucl.ac.uk/spm/software/spm8). For each subject and each task, the data was slice time corrected and smoothed using a full width half maximum (FWHM) Gaussian kernel of 5 mm, with a high pass temporal filter of 128 s applied. Individuals T1 scans were then co-registered and normalized to Montreal Neuroscience Institute (MNI) template space.

A standard two-stage random effects model was used for statistical analysis, firstly with *t*-weighted contrast images comparing significant blood oxygen level-dependent (BOLD) activation, convolved with a standard hemodynamic response function (HRF), for the active condition to the baseline. Secondly, random effects group analyses were conducted. For each WM task, a standard *t*-test was conducted. No clusters were significant at a family wise error (FWE) corrected threshold of *p* < 0.05, therefore a *t*-test with an uncorrected threshold of *p* < 0.01 was conducted, and significant clusters were selected with a feature selecting threshold of FWE corrected *p* < 0.05. Each significant cluster was localized using the SPM Anatomy Toolbox (Version 1.8) [Computer Software] (Eickhoff et al., 2005). Further random effects analyses contrasting the two tasks were conducted to identify any areas where the BOLD response was greater for the maintenance plus manipulation task than the maintenance-only task.

## Results

### Behavioral results

Of the 16 participants, one participant was removed from the maintenance plus manipulation task data as she was unable to complete the task in the scanner. Four participants reported using a primarily “verbal” strategy to rotate the 3-D block shapes, while the remaining 12 reported using a primarily “visual” strategy. The group means, standard deviation and range for subjective difficulty rating, task accuracy and task reaction time are presented in Table 1.

**Table 1:** **Mean, standard deviation and range of reaction time and accuracy results from each WM task presented in MRI scanner**.

**Task**		***M***	***SD***	***SE***	***Min***	***Max***
Maintenance-only (*n* = 16)	Reaction Time (secs)	0.86	0.26	0.06	0.09	1.27
	Accuracy	0.78	0.15	0.04	0.46	0.93
	Difficulty	2.13	0.81	0.2	1	4
Maintenance plus manipulation (*n* = 15)	Reaction Time (secs)	0.98	0.20	0.05	0.70	1.31
	Accuracy	0.70	0.19	0.05	0.39	1
	Difficulty	4.13	1.06	0.27	2	5

The subjective ratings of difficulty were significantly different, with participants reporting the maintenance plus manipulation task to be significantly more difficult (*t* = 4.74, *p* < 0.001, *Cohen's d* = 2.30). There were no significant differences between the two tasks for accuracy or reaction time, however the effect size indicates a moderate difference between accuracy of the two tasks *(t* = 1.79, *p* = 0.095, *Cohen's d* = 0.47) indicating participants performed better on the maintenance-only task. The relationship between accuracy, reaction time and subjective difficulty rating for each task was assessed using a Spearman's Rho due to a mix of both interval and ordinal data. A significant relationship between both tasks was found for reaction time (*r* = 0.58, *p* = 0.03) and accuracy (*r* = 0.615, *p* = 0.02). Subjective difficulty ratings and reaction time for the maintenance-only task were significantly related (*r* = 0.503, *p* = 0.05), while subjective difficulty ratings and accuracy for the maintenance plus manipulation task were significantly correlated (*r* = 0.539, *p* = 0.04). No other significant correlations were found.

### fMRI results

**Figure 2:**
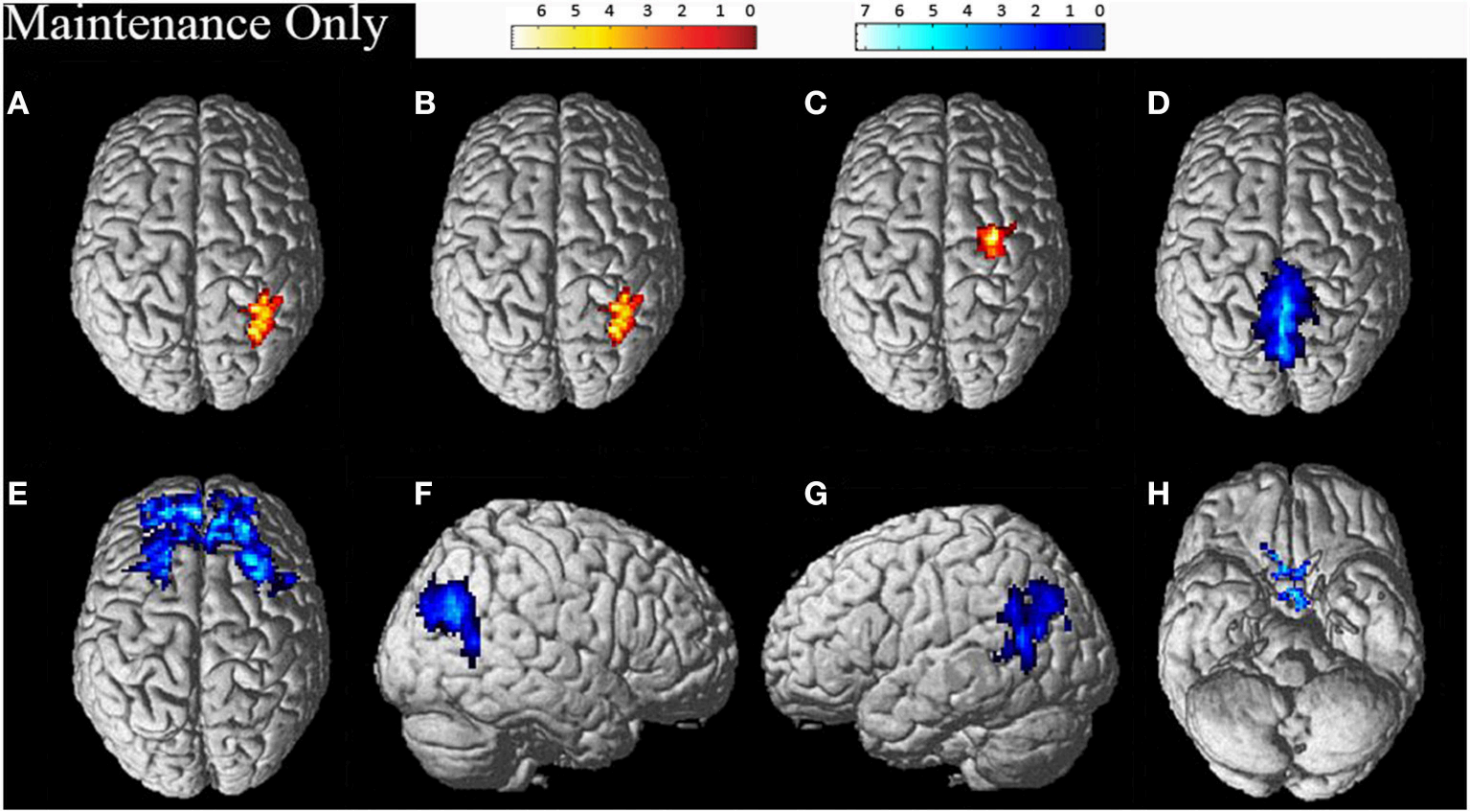
**Significant clusters for the maintenance-only task related activation (displayed in red: A–C) and deactivation (displayed in blue: D–H)**. The cluster labels correspond to the details reported in Table 2.

Individual data was preprocessed and analyzed in terms of active compared to baseline blocks for each task. Random effects group analyses were conducted at an uncorrected threshold of *p* < 0.01, with clusters significant at a FWE corrected threshold of *p* < 0.05, for the maintenance-only task, reported in Table 2. Significant clusters were localized using the Anatomy Tool box version 18 (Eickhoff et al., 2005), with peak cluster coordination reported, and sub-clusters listed below. Corresponding images of significant clusters reported are displayed in Figure 2.

**Table 2:** **Significant clusters at corrected threshold of FWE *p* < 0.05 reported for the maintenance-only *n*-back task (no rotation): task related activation and deactivation**.

**Location**	***k***	***T***	***Z***	**MNI coordinates**		
				**x**	**y**	**z**
**TASK RELATED ACTIVATION**						
(a) Right SPL^*^	553	6.30	4.27	34	−64	50
Right IPL				38	−44	40
Right angular gyrus				34	−56	44
Right IPL				32	−48	42
Right IPL				38	−52	42
(b) Right SMA	418	6.08	4.18	12	14	46
Right SMA				14	4	52
Left SMA				−4	6	58
Left superior frontal gyrus				−12	18	42
Left SMA				−2	16	54
Left SMA				−8	16	44
(c) Right superior frontal gyrus^*^	447	5.39	3.90	26	−8	58
Right BA 6				22	−8	52
Right BA 6				24	−10	54
Right BA 6				28	−10	48
Right BA 6				28	−18	48
Right precentral gyrus				32	−16	54
**TASK RELATED DEACTIVATION**						
(d) Left precuneus^*^	4904	8.52	4.98	0	−44	38
Left MCC				−6	−42	38
Left precuneus				0	−72	38
Left MCC				−10	−48	34
Left PCC				−6	−50	30
Right precuneus				4	−46	44
(e) Right middle occipital gyrus^*^	800	6.76	4.44	38	−82	32
Right angular gyrus				50	−70	36
Right middle temporal gyrus				60	−58	12
Right middle temporal gyrus				40	−68	22
Right middle occipital gyrus				48	−78	26
Right middle occipital gyrus				40	−78	38
(f) Right middle frontal gyrus^*^	4324	6.65	4.40	34	32	48
Left superior frontal gyrus				−26	32	34
Right superior frontal gyrus				24	46	22
Right superior frontal gyrus				28	40	48
Right superior frontal gyrus				26	42	44
Right superior frontal gyrus				22	42	50
(g) Left angular gyrus	757	4.65	3.56	−60	−58	26
Left middle occipital gyrus				−44	−74	38
Left middle temporal gyrus				−56	−66	22
Left middle temporal gyrus				−56	−62	16
Left angular gyrus				−44	−72	42
Left middle temporal gyrus				−48	−52	10
(h) Right Caudate Nucleus	260	4.21	3.33	0	4	0
Right caudate nucleus				6	16	0
Left caudate nucleus				−2	14	−2
Right caudate nucleus				10	22	−2
				12	24	−4
				6	0	0

Cluster size (k-value), T-value, Z-value, and MNI coordinates reported for each cluster identified.

BA, Brodmann's Area; SPL, Superior parietal lobule; IPL, Inferior parietal lobule; SMA, supplementary motor area; MCC, middle cingulate cortex.

* Indicates cluster significant also when analysis is thresholded at p < 0.001 uncorrected. Clusters h had peak voxels in unidentifiable areas and were named in relation to the nearest identifiable location.

**Figure 3:**
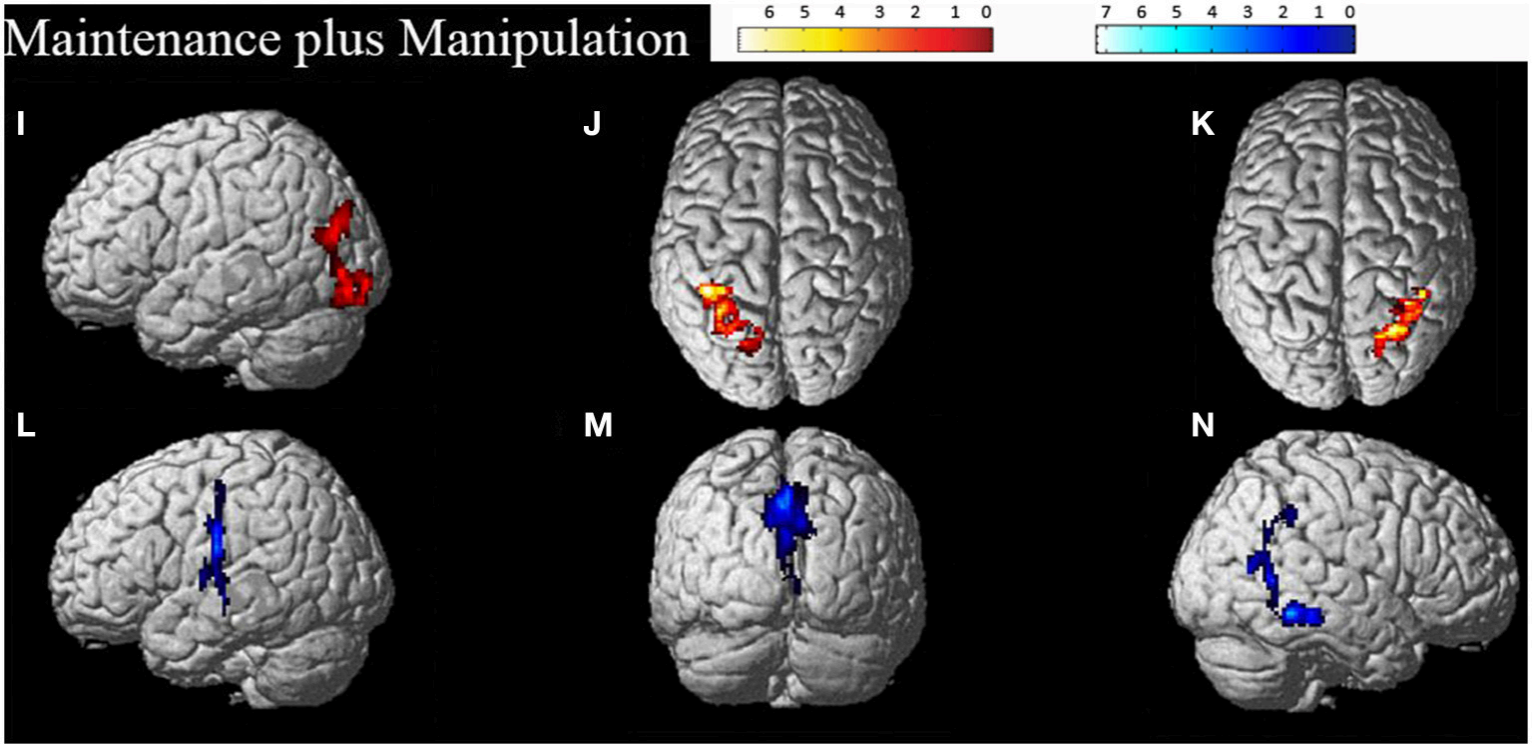
**Significant clusters for the maintenance plus manipulation task related activation (displayed in red: I–K) and deactivation (displayed in blue: L–N)**. The cluster labels correspond to the details reported in Table 3.

Random effects group analyses were also conducted for the maintenance plus manipulation task, also at an uncorrected threshold of *p* < 0.01, with clusters significant at a FWE corrected threshold of *p* < 0.05, reported in Table 3. Significant clusters were localized using the Anatomy Toolbox version 18 (Eickhoff et al., 2005), with peak cluster coordination reported, and sub-clusters listed below. Corresponding images are displayed in Figure 3.

**Table 3:** **Significant clusters at corrected threshold of FWE *p* < 0.05 reported for the maintenance plus manipulation *n*-back task (including rotation): task related activation and deactivation**.

**Location**	***k***	***T***	***Z***	**MNI coordinates**		
				**x**	**y**	**z**
**TASK RELATED ACTIVATION**						
(i) Left Fusiform Gyrus	620	5.63	3.78	−38	−80	−18
Left Inferior Occipital Gyrus				−28	−96	−6
Left Middle Occipital Gyrus				−32	−80	20
Left Middle Occipital Gyrus				−30	−80	24
Left Middle Occipital Gyrus				−26	−82	24
Left Lingual Gyrus				−28	−94	−16
(j) Left IPL	713	5.39	3.69	−36	−48	24
Left SPL				−26	−62	50
Left SPL				−34	−62	54
Left Precuneus				−16	−72	60
Left IPL				−38	−44	48
(k) Right IPL	578	5.01	3.54	38	−50	28
Right Angular Gyrus				36	−60	49
Right Superior Occipital Gyrus				28	−64	40
				42	−44	22
Right Superior Occipital Gyrus				26	−62	30
**TASK RELATED DEACTIVATION**						
(l) Left Postcentral Gyrus	353	8.06	4.52	−54	−18	20
Left Postcentral Gyrus				−64	−16	16
Left Postcentral Gyrus				−46	−16	28
Left Postcentral Gyrus				−62	−16	32
Left Postcentral Gyrus				−60	−14	30
Left Postcentral Gyrus				−48	−18	32
(m) Left MCC	1325	7.03	4.25	−6	−38	40
Left Precuneus				0	−54	50
Left Precuneus				−8	−48	38
Right PCC				12	−46	30
Right MCC				10	−48	34
Left Precuneus				0	−58	28
(n) Right Middle Temporal Gyrus	367	6.89	4.20	62	−34	−14
Right Inferior Temporal Gyrus				56	−44	−12
Right Inferior Temporal Gyrus				58	−46	−14
Right SMG				64	−46	38
Right Middle Temporal Gyrus				60	−60	20
Right SMG				62	−44	42

Cluster size (k-value), T-value, Z-value, and MNI coordinates reported for each cluster identified.

IPL, Inferior parietal lobule; SPL, Superior parietal lobule; PCC, posterior cingulate cortex; MCC, middle cingulate cortex; SMG, supramarginal gyrus.

Between groups *t*-tests contrasting the two tasks were conducted to locate areas which increased in activation for the maintenance plus manipulation task compared with the maintenance-only task, however no significant results were found.

## Discussion

As predicted, consistent with previous research (Wager and Smith, 2003; Owen et al., 2005; Rottschy et al., 2012), the maintenance-only task recruited activation within the fronto-parietal areas. Activation was also largely right lateralized, again consistent with previous research (Vogel et al., 2003; Owen et al., 2005; van der Ham et al., 2009). Activation was revealed in the right superior frontal gyrus, consistent with spatial WM research (Jackson et al., 2011). Activation was also revealed in the right supplementary motor area (SMA). SMA activation has been implicated in feeling and planning (rehearsal for) pressing a button for the task (Tanji, 1994), however as all participants had the button under their right hand, the ipsilateral activation in the right SMA as opposed to the left SMA is surprising. Previous research has suggested left SMA activation may be involved in rehearsal processes in verbal WM (Smith et al., 1996; Nakajima et al., 2014), while bilateral SMA activation in WM tasks has also been seen independent of rehearsal processes (Awh et al., 1996).

However, the largest cluster was found in right SPL, which could reflect the area's involvement in mental rotation (Jordan et al., 2001; Just et al., 2001; Halari et al., 2006; Olson and Berryhill, 2009), manipulation (Champod and Petrides, 2010), attention (Corbetta and Shulman, 2002; Behrmann et al., 2004), and spatial WM (Culham and Kanwisher, 2001; Wager and Smith, 2003; Harrison et al., 2010). More generally, this highlights the importance of the parietal contribution to the task. Surprisingly, no significant activation in the left IPL was revealed, in contrast with our predictions. However, a trend toward significance was found in the left SPL, which suggests bilateral parietal areas may be involved in the task but with the LH involved to a lesser extent. Furthermore, no significant clusters were revealed outside the fronto-parietal areas, consistent with Owen et al. (2005).

The maintenance plus manipulation task revealed heavily bilateral parietal activation as predicted. The right lateralized parietal activation was to be expected considering the spatial demands of the task and is consistent with previous research (Vogel et al., 2003; Wager and Smith, 2003; van der Ham et al., 2009). The largest cluster, however, was in the left IPL. The bilateral parietal activation seen with increased cognitive demand may reflect an increase in attentional resources required to perform the task (Corbetta and Shulman, 2002; Behrmann et al., 2004), and is consistent with the findings of Champod and Petrides (2010) that with added manipulation in a WM task, activation becomes more concentrated in the parietal cortex. Furthermore, on visual inspection of the cluster size (*k*-values, as in Tables 2, 3) in task related activation, the size of the clusters found in the maintenance plus manipulation task all appeared larger than the largest clusters found in the maintenance-only task. This suggests that the added demand of manipulation requires greater effort and attention, and draws on more neural resources to the extent of engaging interhemispheric networks.

Activation during the mental rotation task was also revealed in the left fusiform gyrus, an area which has previously been established to be involved in face encoding tasks (Kanwisher et al., 1997), and has also been shown to increase in activation with increased *n* (demand) in a face-recognition *n*-back task (Druzgal and D'Esposito, 2001). However, the left fusiform gyrus has also been shown to respond during verbal WM retention and letter presentation, but not during imagery or visuo-spatial maintenance in match to sample WM tasks (Hamamé et al., 2012). The activation of the left fusiform gyrus during the maintenance plus manipulation task may reflect a verbal rehearsal strategy for visuo-spatial stimuli rather than just visualization alone (Haxby et al., 1995). Interestingly though, the majority of participants in this study reported using a visual strategy for rotating the shapes. It is possible that activation within this area of the fusiform gyrus serves as an object or categorical representation area, maintaining the stimulus, while the parietal areas manipulate the shapes spatially, consistent with previous parietal research (Culham and Kanwisher, 2001; Jordan et al., 2001; Just et al., 2001; Wager and Smith, 2003; Halari et al., 2006; Olson and Berryhill, 2009; Champod and Petrides, 2010; Harrison et al., 2010).

Of particular interest with regard to the maintenance plus manipulation task is the bilateral activation found in the IPL. The increase in IPL activation with increased difficulty is consistent with the findings of Harrison et al. (2010) who utilized memory load to increase the difficulty of the task, while the current study utilized manipulation of stimuli (mental rotation) to increase difficulty.

Bilateral activation within the parietal lobe has previously been demonstrated in studies exploring mental rotation outside of WM (Halari et al., 2006), although a RH advantage is more commonly demonstrated for simple spatial mental rotation tasks (Corballis, 1997; Jordan et al., 2001; Just et al., 2001; Olson and Berryhill, 2009). It is possible that this may reflect the two hemispheres performing separate processes concurrently, such as LH areas subserving categorical WM, and allocating categories or names for each block shape, during RH performance of more spatial processes (van der Ham et al., 2009). However, if this were the case then it should follow that stronger left IPL activation would be apparent in the maintenance-only task. In either case, it is clear that both WM tasks recruited networks of sites which interact differently depending on the task demands.

One major limitation to the current study is the timing constraints of fMRI studies, with the hemodynamic response (HDR) delay and individual variability (Aguirre et al., 1998; Steffener et al., 2010). While the results of this study allowed us to achieve our aim of localizing the areas predominantly involved in the task, interpretation is limited by being unable to examine timing of activation with greater precision. It is crucial to examine the relationship between the areas activated and the timing of activation, in order to understand how these areas work together and separately in performing WM tasks. Therefore, further using more temporally sensitive techniques such as magnetoencephalography (MEG) or electroencephalography (EEG) would be a beneficial next step toward elucidating the underlying neural mechanisms of WM.

Another issue is apparent in the localization of areas activated using the Anatomy Toolbox (Eickhoff et al., 2005). When comparing the areas of activation displayed in Figures 2, 3 with the corresponding cluster location labels in Tables 2, 3 (respectively), there were four clusters (b, d, e, h) which appeared to be bilateral. However, as the Anatomy Toolbox localizes clusters based on the peak voxel location without considering surrounding statistically significant activity, this may skew the interpretation of the involvement of these areas. Therefore, all sub-clusters were also listed in Tables 2, 3. Furthermore, clusters h, j and k had peak voxels in unidentifiable locations according to the Anatomy Toolbox, and were therefore referred to be localized in the next closest identifiable cluster.

Finally, the use of strategy type adopted to perform the task is important to consider in interpreting these results. As only four of the 16 participants reported using a verbal strategy to rotate the blocks, statistical comparison based on this small group size could not be reliably justified. A more balanced comparison may allow further insight as to whether the LH activation represents categorical WM, while the RH represents more spatial processes, consistent with van der Ham et al. (2009) or may delineate whether the left fusiform gyrus activation reflects a verbal rehearsal process for visuo-spatial stimuli rather than just visualization (Haxby et al., 1995). Participants' strategy type should be taken into consideration for future studies of WM in combination with mental rotation or other forms of stimuli manipulation.

In conclusion, this study adds to the body of evidence implicating networks of fronto-parietal sites in WM activation. While the spatial stimuli remained constant and could be maintained when no rotation was required, the predictable RH activation of fronto-parietal sites was apparent. However, with the added demand of manipulation in the WM task, significant activation was less reliant on frontal areas and a shift toward bilateral IPL and left fusiform gyrus was revealed. This may suggest that simple maintenance and decision making utilizes the frontal areas, but when manipulation is also required, the left fusiform gyrus is required to maintain a categorical representation of the stimulus, while the parietal areas concurrently manipulate the stimulus and direct attention toward goal-directed behaviors. When the results from both tasks are taken into consideration with the networks explored by Menon and Uddin (2010), it is clear that activation within the central executive network and task relative deactivation in the DMN are important for cognitive tasks such as this visuo-spatial WM task. However, significant activation of the sites involved within these networks change with increased demand, relying less on frontal areas and more on parietal areas. Future research should compare these results with tasks engaging non-spatial stimuli maintenance concurrently with a form of manipulation. For example, maintaining facial identity while concurrently having to recognize emotion. It would also be beneficial to take into considering the strategy and time participants use to perform aspects of the tasks, and to combine fMRI data with that from more temporally sensitive imaging modalities such as MEG and EEG.

## Author contributions

GL was the primary researcher, involved in all aspects of collection of data, data analysis, data interpretation, and writing up. BA was involved in data collection and writing up. RL was involved in statistical analysis and writing up. DC was a co-supervisor of GL, oversaw data collection, involved in data analysis and writing up. SC was the primary supervisor of GL, oversaw the entire project, data interpretation, and was involved in writing up.

### Conflict of interest statement

The authors declare that the research was conducted in the absence of any commercial or financial relationships that could be construed as a potential conflict of interest.
